# Proliferation of Resident Macrophages Is Dispensable for Protection during *Giardia duodenalis* Infections

**DOI:** 10.4049/immunohorizons.1900041

**Published:** 2019-08-27

**Authors:** Marc Y. Fink, Jenny Maloney, Aleksander Keselman, Erqiu Li, Samantha Menegas, Christopher Staniorski, Steven M. Singer

**Affiliations:** Department of Biology, Georgetown University, Washington, DC 20057

## Abstract

Infection with the intestinal parasite *Giardia duodenalis* is one of the most common causes of diarrheal disease in the world. Previous work has demonstrated that the cells and mechanisms of the adaptive immune system are critical for clearance of this parasite. However, the innate system has not been as well studied in the context of *Giardia* infection. We have previously demonstrated that *Giardia* infection leads to the accumulation of a population of CD11b^+^, F4/80^+^, ARG1^+^, and NOS2^+^ macrophages in the small intestinal lamina propria. In this report, we sought to identify the accumulation mechanism of duodenal macrophages during *Giardia* infection and to determine if these cells were essential to the induction of protective *Giardia* immunity. We show that F4/80^+^, CD11b^+^, CD11c^int^, CX3CR1^+^, MHC class II^+^, Ly6C^−^, ARG1^+^, and NOS2^+^ macrophages accumulate in the small intestine during infections in mice. Consistent with this resident macrophage phenotype, macrophage accumulation does not require CCR2, and the macrophages incorporate EdU, indicating in situ proliferation rather than the recruitment of monocytes. Depletion of macrophages using anti-CSF1R did not impact parasite clearance nor development of regulatory T cell or Th17 cellular responses, suggesting that these macrophages are dispensable for protective *Giardia* immunity.

## INTRODUCTION

*Giardia duodenalis* (syn. *G. intestinalis, G. lamblia*) is one of the most common intestinal parasites in the world. *Giardia* is spread through fecal-oral transmission with infections initiated through the ingestion of cyst-contaminated drinking water or food. As such, disease burdens are highest in regions where access to clean water is limited. Recently, the Malnutriotion and Enteric Dysfunction (MAL-ED) study found that subclinical *Giardia* infections were associated with childhood stunting yet not necessarily correlated to symptomatic diarrhea; thus, infections with *Giardia* contribute to childhood malnutrition (1). Paradoxically, *Giardia* is also associated with a reduced risk of moderate-to-severe diarrhea (2–5). Acute infections with this noninvasive protozoan can manifest with symptoms typical of intestinal distress or be subclinical. In addition, long-term effects such as postinfectious irritable bowel syndrome and chronic fatigue syndrome have been reported (6–10). Adding to this intrigue, symptomatic patients may not exhibit mucosal inflammation despite having diarrhea, diffuse shortening of the intestinal microvilli, absorptive deficiencies, and epithelial barrier disruption (11–14). The causes for this variation in clinical manifestations and outcomes are still poorly understood, but the host immune response has been reported to play a role in the symptoms associated with *Giardia* infection. Previous work from our laboratory suggests that T cells are critical for clearance of *Giardia* infections (15, 16) and also contribute to immunopathology (17). Recent work has also shown that IL-17 is the major T cell cytokine necessary for protective immunity against *Giardia* (18–22). However, it is unclear which innate cells are responsible for the initial recognition of the *Giardia* parasite and the activation of T cells.

Myeloid cells, such as macrophages and dendritic cells, function as initiators of immunity through the use of pattern recognition receptors and also activators of T cell proliferation and differentiation. Dendritic cells and their importance to protective *Giardia* immunity has been described, as these cells are a significant source of IL-6 during *Giardia* infections, and infected IL-6–deficient mice have a defect in parasite clearance (23–25). In contrast, the role of macrophages has not been as well studied, despite their ability to recognize pathogens and contribute to activation of downstream immune responses. Moreover, little work has been done to understand the role of macrophages in vivo during *Giardia* infections.

Our laboratory has previously identified an increase in a population of intestinal macrophages following *G. duodenalis* infection that expresses both NO synthase 2 (NOS2) and arginase 1 (ARG1) (26), markers that are used to distinguish between canonical macrophage subsets: inflammatory M1 (NOS2^+^) and regulatory M2 (ARG1^+^) macrophages. Similarly, following *G. muris* infections in IL-10–deficient mice, there is an expansion of colonic CD11b^+^, CD11c^−^ macrophages that resulted in the inflammation of the colon, suggesting that macrophages have a role in downregulating inflammatory signals in wild-type (WT) mice (27). In murine primary cell cultures, thioglycollate-derived peritoneal macrophages also were reported to release extracellular traps in response to *Giardia* trophozoites to ensnare parasites (28), and human macrophages are also reported to be able to phagocytize *Giardia* trophozoites (29–31). However, beyond these reports, it is unclear if macrophages have an important influence on *Giardia* immunity. As such, the goals of this study were to further define the phenotype of accumulating duodenal macrophages, to determine the mechanism of macrophage accumulation during infection, and to assess the role of these cells in *Giardia* immunity as possible activators of T cells or as effector cells contributing to control of the infection.

In this study, we further define the phenotype of accumulating macrophages to be F4/80^+^, CD11b^+^, CD11c^int^, CX3CR1^+^, MHC class II^+^ (MHCII^+^), Ly6C^−^ cells that express both ARG1 and NOS2. We also show that macrophage accumulation was not impeded in CCR2^−/−^ mice and, instead, 5-ethynyl-2′-deoxyuridine (EdU) proliferation studies revealed that tissue-resident macrophages accumulate within the intestine postinfection. Last, we show that depletion of macrophages does not inhibit parasite clearance and that induction of critical Th17 cellular responses were not impacted, suggesting that macrophages are dispensable for protection.

## MATERIALS AND METHODS

### Parasites culture, infections, and counts

*G. lamblia* (strain GS/M/H7) was obtained from American Type Culture Collection, Manassas, VA (catalog no. 50581). Parasites were cultured in standard TYI-S-33 medium supplemented with bovine bile, l-cysteine, ascorbic acid, and an antibiotic-antimycotic solution (Sigma-Aldrich, St. Louis, MO). In preparation for infection, *Giardia* trophozoites were thoroughly washed in PBS for several cycles and then resuspended in PBS. Mice were infected via gavage with 1 × 10^6^ *Giardia* trophozoites in 0.1 ml of PBS. Following euthanasia, parasites were counted from intestinal segments extracted from infected animals by icing duodenum segments (3–5 cm immediately distal to the ligament of Treitz) for 30 min in PBS. Intestinal sections were then minced, and trophozoites were counted on a hemocytometer.

### Mice

C57BL/6J, CCR2^−/−^ (B6.129S4-Ccr2^tm1Ifc^/J) and CX3CR1-GFP (B6.129P2(Cg)-*Cx3cr1*^*tm1Litt*^/J) mice were obtained from The Jackson Laboratory (Bar Harbor, ME) and kept under specific pathogen-free conditions at Georgetown University. CX3CR1-GFP were bred at Georgetown University, and all littermates were used for comparative infection experiments. All experiments were performed with the approval of the Georgetown University Animal Care and Use Committee.

### Isolation of intestinal lamina propria cells

Intestinal segments from individual mice were digested to liberate cells residing within the duodenal lamina propria of the small intestine (32). Briefly, segments were excised, Peyer patches removed, and intestinal segments were opened longitudinally. Segments were then minced into small segments and washed in complete medium (CM) (HBSS supplemented with 2% FBS and 1× antibiotic-antimycotic solution) with 1 mM DTT for 15 min at 37°C within a shaking incubator to remove luminal mucous. Segments were then washed with CM and 1.3 mM EDTA as before to remove epithelial cells. Tissues were washed three times with CM and then resuspended in RMPI 1640 supplemented with 2% FBS, 1× antibiotic-antimycotic solution, 1 mg/ml type IV collagenase, and 0.5 mg/ml DNAse (digestion medium) and digested at the same speed and temperature with shaking to digest tissue. Finally, tissues were passed through a 40-μm cell strainer in preparation for staining. If intestinal segments were pooled for digestion, the pooled duodena were fractionated to collect lamina propria cells as described (33). After enzymatic digestion, the lamina propria fraction was separated on a Percoll (Sigma-Aldrich) gradient to enrich for leukocytes and remove dead cells.

### Flow cytometry

Lamina propria cell preparations were washed in PBS, and cells were stained with LIVE/DEAD Fixable Violet Stain (Invitrogen) for 30 min at 4°C in the dark. Cells were FcR blocked with TruStain FcX (anti-mouse CD16/32 Ab) (BioLegend) for 15 min at room temperature in the dark and then immediately stained for cellular surface markers with fluorophore-conjugated Abs against F4/80, CD11b, CD11c, MHCII, Ly6C, CD3, CD4, and CD8 (all BioLegend) for 20 min at 4°C. If staining for intracellular proteins, cells were fixed and permeabilized using the True-Nuclear Transcription Factor Buffer Set (BioLegend). Intracellular staining with fluorophore-conjugated Abs ARG1 (R&D Systems), NOS2 (Santa Cruz Biotechnology), RORγt, Foxp3 (eBioscience), and T-bet (BioLegend) was performed for 1 h at room temperature. Isotype-matched control Abs were tested simultaneously for all stains. Stained cells were analyzed using a FACStar Plus dual laser system (Becton Dickinson) and FCS Express version 6.0 software (DeNovo Software).

### EdU labeling

C57BL/6J mice received EdU in drinking water at a concentration of 0.2 mg/ml from days 3 to 7 postinfection. Drinking bottles were covered in foil to prevent light-mediated degradation, and EdU water was replenished at day 5. The incorporation of EdU into small intestinal lamina propria cells was assessed using the Click-iT Plus EdU Alexa Fluor 647 Flow Cytometry Assay Kit (Invitrogen) per the manufacturer’s protocol. All other Ab staining was done as previously described.

### Macrophage depletion studies

Anti-CSF1R (CD115) mAb (clone AFS98) (Bio X Cell, Lebanon, NH) was used to deplete macrophage populations in C57BL/6J mice, and control mice received anti-trinitrophenol IgG2a isotope control Ab (clone 2A3) (Bio X Cell). All mice received 1.0 mg of control or depletion Ab on day −1 and 0 (infection day); mice then received 0.5 mg of Ab on days +3 and +5.

### Real-time PCR

Small intestinal tissue was collected from all groups following the 7-d infection. Segments were homogenized in TRIzol (Invitrogen) and total RNA extracted following the manufacturer’s protocol. RNA (500 ng input) from each sample underwent gDNA treatment and was then reverse transcribed to cDNA using an iScript gDNA Clear cDNA Synthesis Kit (Bio-Rad) following the manufacturer’s protocol. Genes were amplified using validated PrimePCR Primers (Bio-Rad) for IL-10 (qmmuced0044967), TNF-α (qmmuced0004141), IL-6 (qmmucid0005613), IL-23p19 (qmmuced0045759), and IL-17A (qmmucid0026592) and expression was normalized to GAPDH (qmmuced0027497). Real-time PCR was performed on cDNA with the primers listed above on a CFX96 cycler (Bio-Rad) using Power SYBR Green PCR Master Mix (Applied Biosystems). All samples were assayed in triplicate and analyzed using the ΔΔ cycle threshold method to assess relative fold change for IL-10, TNF-α, IL-6, IL-23p19, and IL-17A.

### Statistical analysis

Statistical significance of flow cytometry gate statistics was determined using either one-tailed or two-tailed *t*-tests. All statistical tests, analyses, and graphs were performed with GraphPad Prism 8.0 software (La Jolla, CA).

## RESULTS

### F4/80^+^, CD11b^+^, CD11c^int^, CX3CR1^+^, MHCII^+^, Ly6C^−^, cells accumulate in the mouse small intestine following infection

**FIGURE 1.**
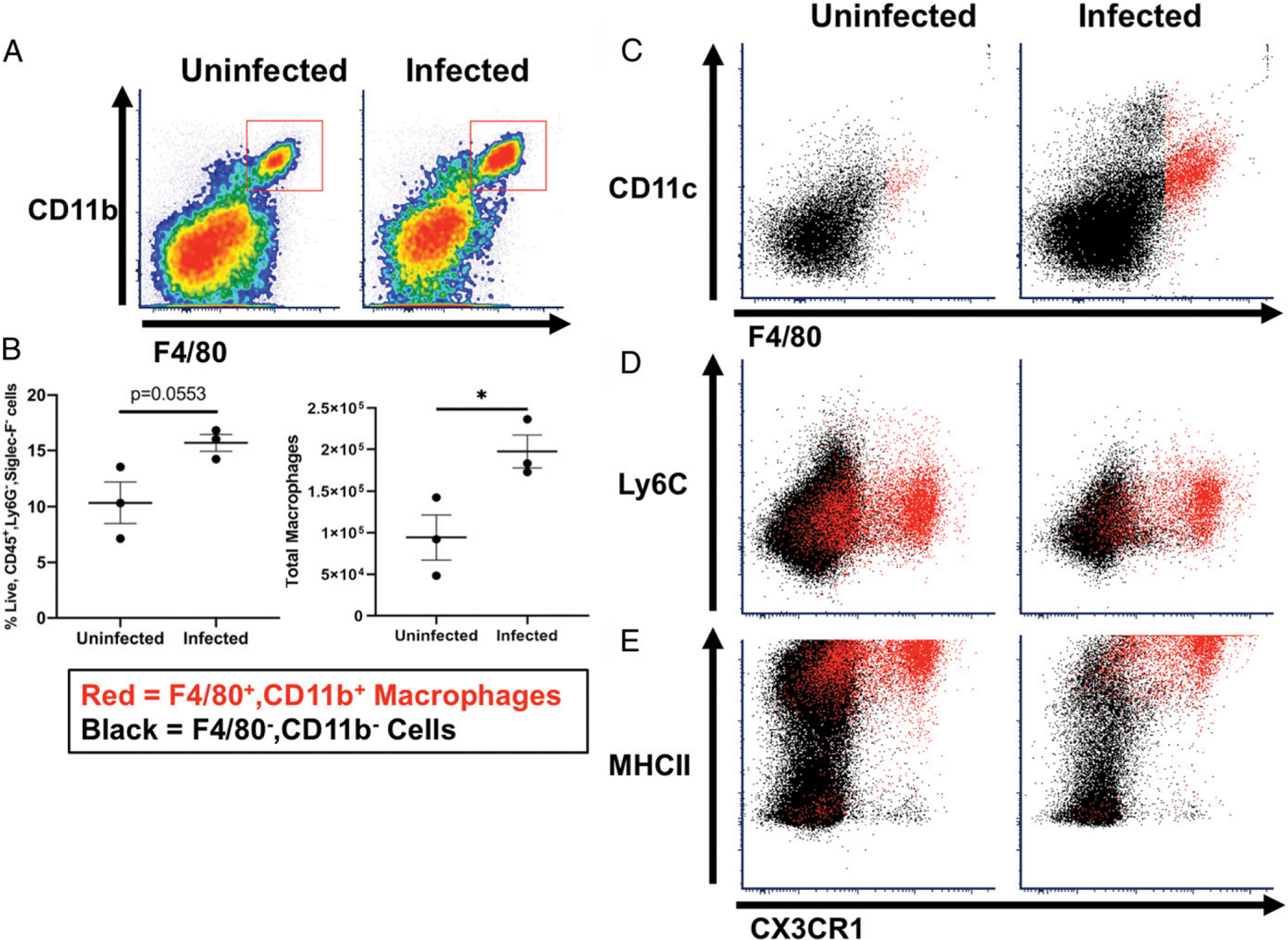
Phenotypic characterization of duodenal macrophage in response to *Giardia* infection. CX3CR1-GFP mice were infected with 1 × 10^6^ *Giardia* trophozoites on day 0 and euthanized on day 7. Staining for F4/80 is shown in combination with CD11b (**A**), CD11c (**C**), Ly6C (**D**), and MHCII (**E**). CX3CR1 expression was assessed through the detection of endogenous GFP. Cells are gated on live, CD45^+^, Ly6G^−^, Siglec-F^−^ cells. (A) F4/80 and CD11b staining was examined on cells from duodenum and jejunum of uninfected and infected mice; and (B) the comparative cell frequencies and total numbers of F4/80^+^, CD11b^+^ macrophages (in the red boxes in (A) are shown for individual mice. (C–E) Surface expression of CD11c (C), Ly6C (D), and MHCII (E) of F4/80^+^, CD11b^+^ macrophages are shown as red dots, with black dots representing F4/80^−^, CD11b^−^ cells. All flow cytometry plots are shown from one individual mouse from the uninfected and infected groups and are representative of three mice per group. Flow cytometry density plots were adjusted with smoothing = 20. Flow cytometry data are representative of two independent experiments using CX3CR1-GFP mice. Error bars represent mean ± SEM. Each circle represents data from one mouse with *p < 0.05 by a two-tailed *t*-test.

**FIGURE 2.**
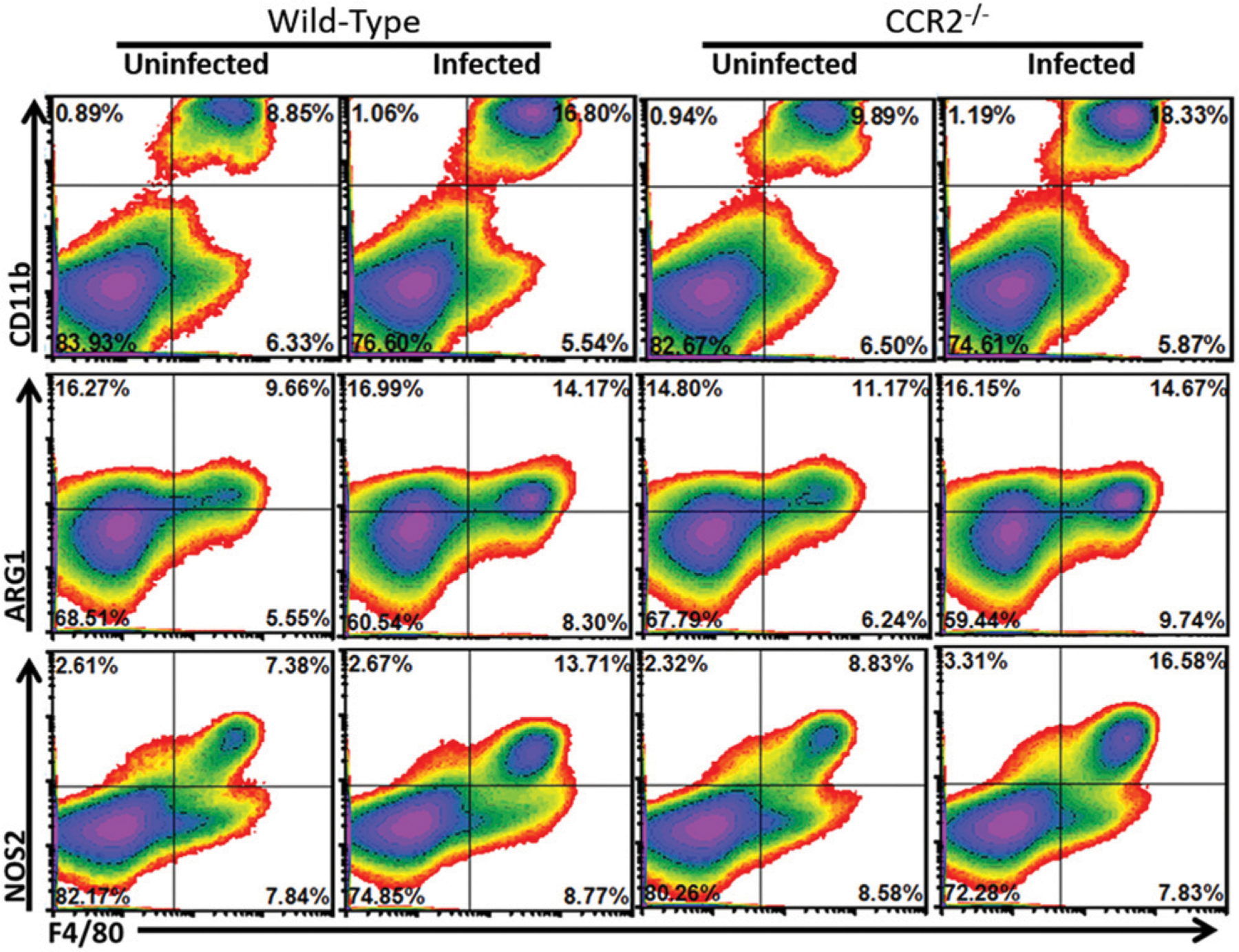
Macrophage accumulation occurs in CCR2^−/−^ mice during infection. Mice were infected with 1 × 10^6^ trophozoites on day 0 and euthanized at day 7. Staining for F4/80 is shown in combination with CD11b, ARG1, and NOS2 in CCR2^−/−^ and WT C57BL/6J mice. Flow cytometry plots represent pooled intestines of four mice per group.

To characterize macrophages of the small intestine during *G. duodenalis* infection, intestinal cells residing in the lamina propria were collected from the duodenum of CX3CR1-GFP (Fig. 1) mice 7-d postinfection and compared with uninfected control mice. The collected cells were stained with Abs againstCD45, Siglec-F,Ly6C, F4/80, CD11b, CD11c, and MHCII to phenotype macrophages and distinguish them from other cells of the myeloid lineage (34). CXC3CR1 was also assessed through the detection of endogenous GFP using CX3CR1-GFP reporter mice. After a 7-d infection, we report a significant increase in the frequency and total cell number of F4/80^+^, CD11b^+^ duodenal macrophages in *Giardia*-infected mice when compared with uninfected mice (Fig. 1A, 1B). When this macrophage population was further examined, flow cytometry revealed that F4/80^+^, CD11b^+^ macrophages additionally express high levels of CX3CR1 (Fig. 1D, 1E) and MHCII (Fig. 1E). Most of these cells also displayed an intermediate level of CD11c expression (Fig. 1C); however, we did detect a minimal increase in CD11c^hi^-expressing macrophages. Additionally, these macrophages also had little to no expression of Ly6C when compared with uninfected control groups (Fig. 1D). We have previously reported an accumulation of ARG1- and NOS2-expressing macrophages in the small intestine during *Giardia* infection (26), and this expression was confirmed in the macrophages described in this study (Fig. 2). The cells described (F4/80^+^, CD11b^+^, CD11c^int^, CX3CR1^+^, MHCII^+^, Ly6C^−^) in this paper are consistent with expansion of resident macrophages (35).

### Macrophages accumulate in the duodenum of CCR2^−/−^ mice during *Giardia* infection

During infections, macrophages are often recruited to the tissue in a CCR2-dependent manner (36, 37). To determine if the macrophages described in this study represent an expansion of resident cells rather than the recruitment of blood monocytes to the intestine, we assessed macrophage accumulation during *Giardia* infection using CCR2-deficient mice. CCR2^−/−^ mice and C57BL/6J WT control mice were infected with *Giardia* trophozoites, and macrophages were collected from the duodenum of mice 7 d postinfection and from uninfected controls. We have previously reported that ARG1, NOS2 dual expressing macrophages accumulate in the intestine following infection with *Giardia* (26) and used these markers to assess accumulation in this experiment. Macrophage accumulation was unaffected by CCR2 deficiency, supporting in situ expansion as the source of these cells (Fig. 2). Parasite burdens were measured from WT and CCR2^−/−^ duodenum at day 7 to determine if CCR2-dependent recruitment is required for parasite clearance; however, no significant difference in parasite burden was detected between CCR2^−/−^ mice and WT mice (data not shown). Collectively, these data indicate that CCR2 expression is not required for macrophage accumulation or parasite clearance during *Giardia* infection.

### Duodenal macrophages proliferate in situ during *Giardia* infection

**FIGURE 3.**
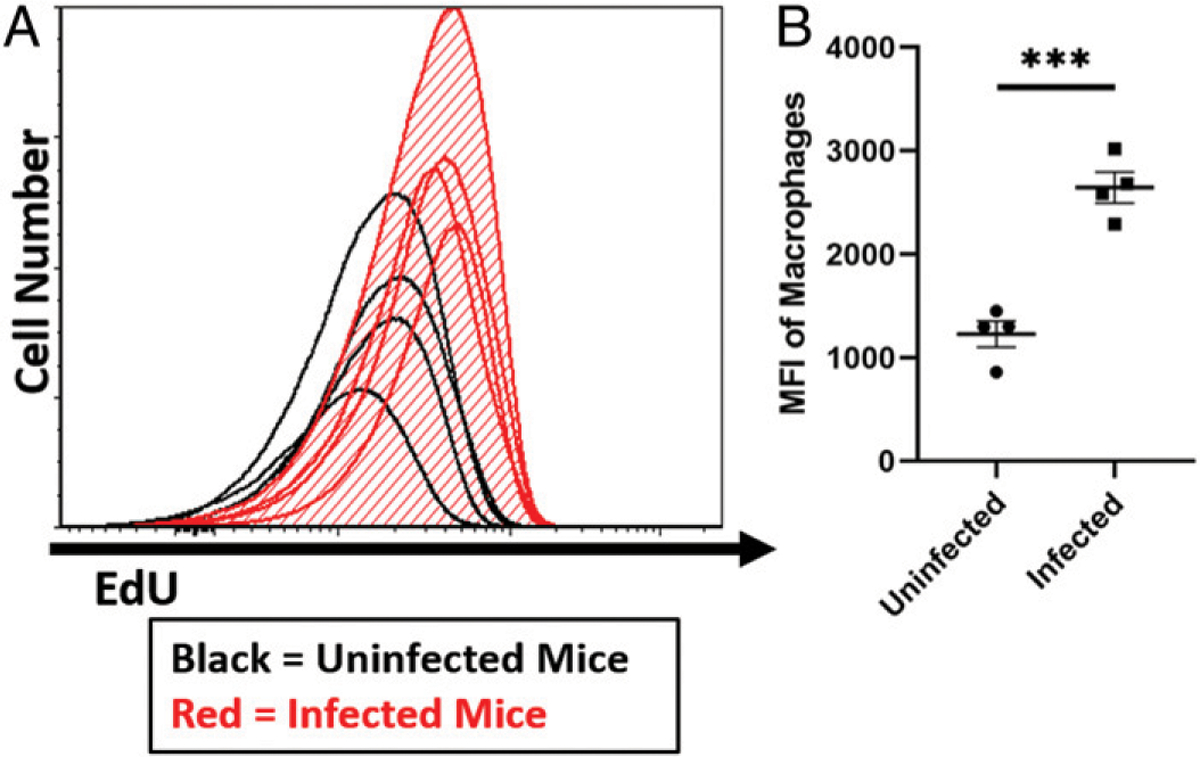
Macrophage accumulation is due to in situ proliferation during *Giardia* infection. WT C57BL/6J mice were infected with 1 × 10^6^ trophozoites on day 0 and euthanized on day 7. An EdU incorporation assay was used to detect proliferation of cells within the intestine. EdU was given at a concentration of 0.2 mg/ml in drinking water from day 3 to 7 postinfection. (**A**) Histogram shows detection of EdU^+^ macrophages using flow cytometry. Each line represents macrophages (CD11b^+^, CD11c^+^, F4/80^+^ cells) from an individual mouse, and uninfected mice are shown in black lines and *Giardia*-infected mice using red lines. (**B**) Mean fluorescence intensities of EdU^+^ macrophages were also compared between both groups. Cells are triple-gated on F4/80^+^, CD11b^+^, CD11c^+^ cells. Infection and proliferation data are representative of two independent trials with four mice per group. Error bars represent mean ± SEM. Each circle and square represents data from one mouse with ***p < 0.0005 by a two-tailed *t*-test.

As CCR2 deficiency did not interfere with macrophage accumulation in this model, we next asked if tissue-resident macrophage proliferation was occurring in response to infection. To determine if resident macrophages proliferate in response to *Giardia* infection, infected mice and uninfected controls were administered EdU. EdU is a thymidine analogue that is incorporated into the DNA of replicating cells and can be used to measure cellular proliferation (38). EdU was added to the drinking water of C57BL/6J mice at a dose 0.2 mg/ml EdU in their drinking water from days 3 to 7. Using flow cytometry analysis, we detected greater incorporated EdU levels and higher numbers of EdU^+^ cells in F4/80^+^, CD11b^+^, CD11c^+^ cells derived from *Giardia*-infected mice when compared with uninfected controls(Fig.3A).Macrophages isolated from the infected group had a significantly greater average mean fluorescence intensity (2643.89 ± 150.02) when compared with uninfected mice (1228.42 ± 127.48) (Fig. 3B). These data indicate that the accumulation of macrophages during *Giardia* infection results from the proliferation of tissue-resident macrophages.

### Depletion of macrophages does not affect infection outcome

**FIGURE 4.**
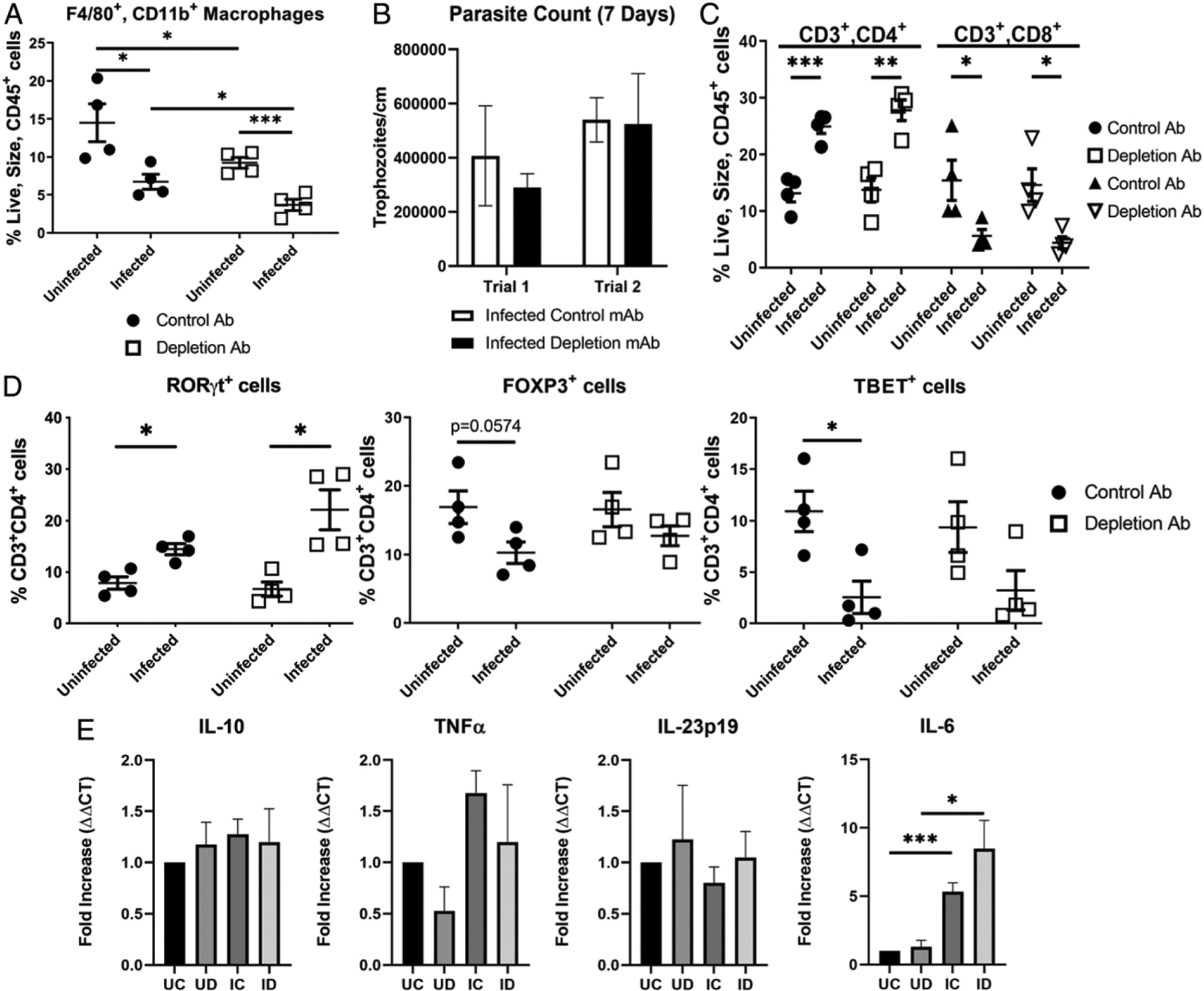
Depletion of murine macrophages does not affect infection outcome. Flow cytometry analysis of intestinal lamina propria–derived (**A**) F4/80^+^, CD11b^+^ macrophages, (**C**) CD3^+^ T cells and (**D**) CD4^+^ Th cell subsets. All immune cells were extracted from uninfected or *Giardia*-infected mice treated with either isotype control Ab (circle, up-facing triangle) or anti-CSF1R depletion Ab (square, down-facing triangle). (**B**) Parasite counts from C57BL/6J mice that were infected with the GS strain of *Giardia* for 7 d and treated with anti-CSF1R depletion Ab or isotype control Ab. Trophozoites were counted from murine duodenum with four mice per group. (E) Gene expression of intestinal cytokines extracted from 1 cm of whole murine duodenum using TRIzol-based RNA purification methods. qRT-PCR was used to examine fold changes of IL-10, TNF-α, IL-23p19 (IL-23a), IL-6, and IL-17A (not detected in uninfected groups) within the duodenum during infection and compared with mice that were uninfected and treated with the isotype control Ab. Bar graphs represent mean ± SEM. Flow cytometry and parasite count data are representative of two independent experiments, and cytokine expression data are from trial 2. Each circle and square represent data from one mouse with (A) *p < 0.05, **p < 0.005, ***p < 0.001 by one-tailed *t*-test and (B–E) *p < 0.05, **p < 0.005, ***p < 0.001 by two-tailed *t*-test.

To determine if tissue macrophages contribute to the development of immune responses to *Giardia*, we depleted macrophages in vivo using a mAb specific for CSF-1R (CD115, CSF1R). CSF-1 is required for the maturation and replacement of tissue-resident macrophages; thus, mice deficient in CSF-1 signaling also exhibit a substantial decrease in F4/80^+^ macrophages in tissues (39–41). C57BL/6J mice were treated with anti-CSF1R or isotype control Ab prior to infection with *Giardia* and throughout the 7-d infection. Flow cytometry analysis revealed reduced numbers of duodenal macrophages at day 7 of infection in mice that had received the depleting Ab (Fig. 4A, Supplemental Fig. 1A); however, parasite burden following infection did not change between mice receiving depleting or control Ab (Fig. 4B). Flow cytometry also revealed an increase in the frequency of CD3^+^, CD4^+^ Th cells (Fig. 4C, Supplemental Fig. 1B) along with a reduction in the frequency of CD3^+^, CD8^+^ cytotoxic T cells in infected groups (Fig. 4C). Macrophage depletions did not affect overall CD3^+^ cell changes between control Ab–treated and depletion Ab–treated groups (Fig. 4C). When compared with other trials (Supplemental Fig. 1), the data for this trial (Fig. 4) did not show an increase in the macrophage population of the infected, control Ab–treated group, and anti-CSF1R depletion did not seem as efficient (Fig. 1A). However, in both trials, no parasite clearance defect was apparent (Fig. 4B) and the proliferation of critical CD4^+^ T helper cells was still induced (Fig. 4C, Supplemental Fig. 1B). Intracellular staining of RORγt, Foxp3, and T-bet transcription factors was conducted to assess any changes to the proportion of Th17, regulatory T cell, and Th1 T cell subsets, respectively, during macrophage depletion. When compared with control mice, infected groups were still able to increase frequency of RORγt^+^ Th17 cells regardless of macrophage depletion (Fig. 4D). Moreover, a decrease in the frequency of regulatory T cells and Th1 cells also occurred when comparing the same groups. This increase in Th17 cells was consistent with a 5-fold increase of IL-6 mRNA levels in the infected control group and an 8. 5-fold increase in the infected depleted group, based on quantitative real-time PCR (qRT-PCR) analysis of whole-gut tissue (Fig. 4E). IL-17A expression was also examined using qRT-PCR, and all *Giardia*-infected group samples amplified during this assay; however, we are unable to report fold increase for IL-17A, as both control Ab and depletion Ab groups for uninfected mice were unable to amplify within 40 cycles, suggesting these samples had little to no IL-17A transcript available. Depletion of macrophages did not significantly affect mRNA levels of IL-10, TNF-α, or IL-23p19 during infection (Fig. 4E). Collectively, these data suggest that macrophages are dispensable for parasite clearance and for the development of T cell responses during *Giardia* infections.

## DISCUSSION

In this report, we sought to determine the mechanism leading to accumulation of duodenal macrophages during *Giardia duodenalis* infection and to determine if these cells were essential to the induction of protective *Giardia* immunity. We show that a population of F4/80^+^, CD11b^+^, CD11c^int^, CX3CR1^+^, MHCII^+^, Ly6C^−^, ARG1^+^, and NOS2^+^ macrophages accumulate in the mouse small intestine during *Giardia* infection. Consistent with a tissue-resident macrophage phenotype, we show that these macrophages accumulate in the small intestine through proliferation rather than the recruitment of monocytes. However, macrophage depletion studies did not impact parasite clearance, and critical Th17 cellular responses were still induced and activated, suggesting that these macrophages are dispensable for protective *Giardia* immunity.

Historically, the separation of macrophages and dendritic cells was based on the expression of F4/80 and CD11c/MHCII; however, it has been demonstrated that within the intestine these cells are able to express either of these canonical markers (35). As every unique myeloid cell phenotype may have a different effector role or represent a particular developmental stage in immunity, it was critical for us to further refine the surface phenotype of accumulating cells. We observe the accumulation of a macrophage population lacking Ly6C, expressing intermediate levels of CD11c and high levels of F4/80, CD11b, MHCII, and CX3CR1. This phenotype is associated with mature resident macrophages rather than phenotypes of recruited monocytes (35, 42). The classification of these cells as resident macrophages is further supported by their expansion in the intestine of *Giardia*-infected CCR2-deficient mice. This model differs from other studies of macrophage recruitment during infection and inflammation. In murine infection models using *Toxoplasma gondii* (43, 44), *Citrobacter rodentium* (45), and HSV-2 (46), Ly6C^hi^ monocytes reportedly accumulate in infected tissues to control infection and do so in a CCR2-dependent manner. These Ly6C^hi^ monocytes then differentiate into Ly6C^−^ macrophages through a process that takes about 5 d to complete. In this model, Ly6C^hi^, MHCII^−^, CX3CR1^int^ monocytes enter intestinal tissue and first increase MHCII expression while gradually decreasing Ly6C expression as they differentiate into macrophages (47–50). These MHCII^hi^ macrophages can then be separated based on CX3CR1 expression, with the larger population of CX3CR1^hi^ cells being the most mature resident gut macrophages (47, 49–51). In murine models of colitis, the overall regulatory effects of CX3CR1^hi^ resident cells are overcome by the inflammatory effects of CX3CR1^int^ cells that are derived from Ly6C^hi^ cells to promote inflammation (49, 50). In contrast to these past reports, our findings suggest that during murine *Giardia* infections, the responding intestinal macrophages do not phenotypically resemble Ly6C^hi^ cells and instead appear to be resident macrophages.

The macrophage population reported in this study was found to be dispensable for parasite clearance during *Giardia* infection. However, these cells may play an important role in immune regulation during infection. Intestinal macrophages are spatially located right beneath the epithelial cell monolayer and are functionally characterized by their highly active phagocytic activity of not only foreign Ag (52) but also host cellular debris such as apoptotic cell bodies (53–55). It is well reported that *Giardia* trophozoites induce apoptosis of host cells during infection. Increased rates of epithelial apoptosis were reported in human biopsy specimens of patients suffering from chronic *Giardia* infections when compared with control samples (56), and in cell culture, human intestinal epithelial cells initiated apoptosis in a caspase-3–dependent manner when exposed to *Giardia* trophozoites (13, 57). Similarly, increased rates of apoptosis were also induced when Caco2 cells were exposed to *Giardia* excretory-secretory products (58, 59). Within this context, rather than supporting protective immunity, it is possible that the accumulation of resident macrophages may be involved in the maintenance of the epithelial barrier through clearance of epithelial cell apoptotic bodies caused by *Giardia*. This clearance and maintenance process is associated with macrophages that exhibit regulatory characteristics (60). An increase in regulatory macrophages could also be a potential mechanism for limiting mucosal inflammation during symptomatic *Giardia* infection.

Intestinal macrophages often display a regulatory role, helping to limit inflammatory responses against intestinal microbes. These cells downregulate many TLR signaling molecules associated with the inflammatory response (61) and increase expression of IL-10 (48, 62) to reduce any inflammatory signals. Our laboratory has previously shown that ARG1^+^, NOS2^+^ macrophages accumulate in the duodenal lamina propria following infection (26) and resemble myeloid-derived suppressor cells (63). An accumulation of these cells has also been reported in other parasitic infection models and functionally are reported to act in an immunosuppressive capacity (64, 65). The expansion of CD11b^+^, CD11c^−^ macrophages during *G. muris* infection in IL-10–deficient mice resulted in the inflammation of the colon, suggesting that macrophages normally have a regulatory role and suppress intestinal inflammation during *Giardia*sis (27). Whether or not the macrophage population reported in this study contributes to immune regulation or the suppression of inflammation remains to be fully elucidated.

Intestinal macrophages may also play a role integrating immune responses among diverse microbes within the intestinal tract. Within the field of *Giardia* immunity, it is quite clear that the intestinal microbiota not only provides protection against this parasite (17, 66, 67) but also more broadly has an effect on the induction of intestinal immune cells (68, 69). Moreover, data from the Global Enteric Multicenter Study (GEMS) suggest that the presence of *Giardia* reduces moderate-to-severe diarrhea during coinfection with other enteric pathogens (2, 4, 5). As resident macrophages are long lived (51), a *Giardia*-induced accumulation of resident macrophages may aid in control of other pathogens or in limiting the disease manisfestations caused by these other pathogens. These mechanisms, however, remain speculative at this point.

MHCII expression in our reported macrophage population was high. Yet these intestinal macrophages did not appear to be effective at the initiation of naive T cell activation. In our studies, the frequency of CD3^+^, CD4^+^ T cells still increased as expected following infection, and various CD4^+^ T cell subsets were similar between control and macrophage-depleted groups. Thus, although naive CD4^+^ T cells appear to be activated normally after macrophage depletion, it remains possible that MHCII expression on these macrophages plays a role in Ag presentation to effector T cells in the gut, which then leads to the maintenance and proliferation of Ag-specific T cells. A previous study with OT-II transgenic T cells and adoptive transfer of CX3CR1^+^ macrophages suggest that MHCII-dependent Ag presentation by resident macrophages is important for aiding in T cell proliferation and differentiation of effector T cell populations, rather than driving differentiation of naive T cells within the lamina propria (70).

Despite the accumulation of macrophages with a potentially regulatory phenotype, our depletion studies suggest that macrophages are not critical players in protective *Giardia* immunity nor do they have any regulatory effect on the induction of T cell differentiation. Importantly, IL-6 production and the differentiation of RORγt^+^ Th17 cells (18–22) both appeared normal in macrophage-depleted mice. This is consistent with our earlier findings that indicate dendritic cell production of IL-6 is critical for initiation of protective anti-*Giardia* immunity (25). It remains to be determined if these macrophages play other roles during Giardiasis, such as limiting or contributing to immunopathogenesis or influencing immune responses during coinfections. Given the importance of intestinal macrophages in the recognition of pathogens and shaping of the host immune response, a better understanding of their role in *Giardia* protective immunity is merited.

## Supplementary Material

Supplemental Material

## Abbreviations used in this article:

ARG1: arginase 1
CM: complete medium
EdU: 5-ethynyl-2′-deoxyuridine
MHCII: MHC class II
NOS2: NO synthase 2
qRT-PCR: quantitative real-time PCR
WT: wild-type
