# Supplementary material for: Proliferation of Resident Macrophages Is Dispensable for Protection during Giardia duodenalis Infections
Source: Immunohorizons. Author manuscript; Available in PMC 2020 Feb 21. (PMC7033283; doi:10.4049/immunohorizons.1900041)
Supplement: Supplemental Material [file NIHMS1559690-supplement-Supplemental_Material.pdf]

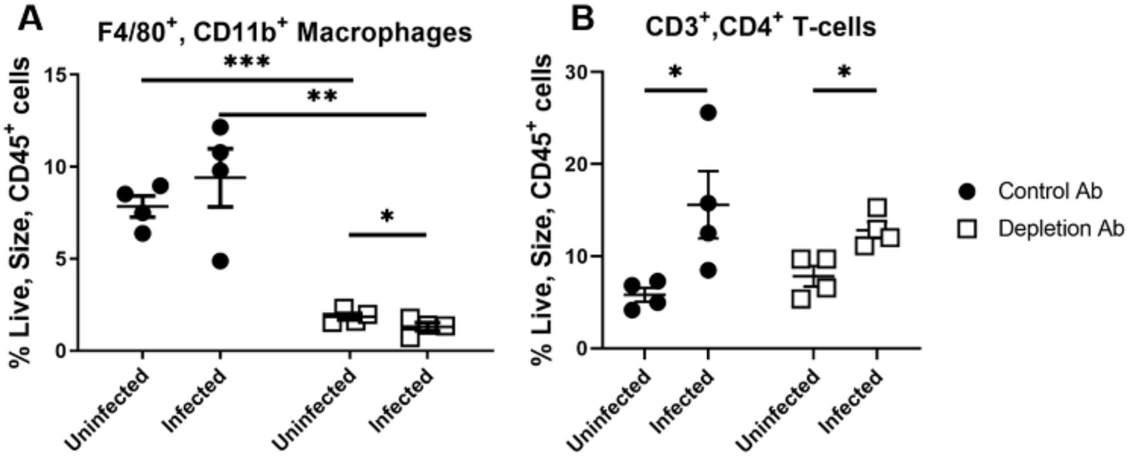

Figure S1: CSF1R monoclonal antibody is capable of depleting murine duodenal macrophages. Flow cytometry analysis of intestinal lamina propria-derived (A) F4/80$^+$, CD11b$^+$ macrophages and (B) CD3$^+$, CD4$^+$ T-cells. Cells were isolated from the lamina propria of the small intestine of uninfected and *Giardia*-infected mice treated with either isotype control Ab (circle) or anti-CSF1R depletion Ab (square). Each circle and square represent data from one mouse with (A) * $P < 0.05$, ** $P < 0.005$, *** $P < 0.001$ by one-tailed t-test and (B)* $P < 0.05$ by two-tailed t-test.
